# Management of Complex Ovarian Cysts in Newborns – Our Experience

**DOI:** 10.21699/jns.v6i1.448

**Published:** 2017-01-01

**Authors:** S Manjiri, SK Padmalatha, J Shetty

**Affiliations:** Department of Pediatric Surgery, M.S. Ramaiah Medical College, Bangaluru-560054, India.

**Keywords:** Ovarian cysts, Newborns, Antenatal ultrasound

## Abstract

Aims: To analyse the clinical presentation, clinicopathological correlation and management of complex ovarian cysts in newborn and infants.

Materials and Methods: Over a period of 6 years (2009-2015), 25 newborns who were diagnosed to have ovarian cyst on antenatal ultrasound, were followed up. We collected data in the form of clinical features, radiological findings, pathology and mode of treatment.

Results: Of the 25 fetuses who were diagnosed to have ovarian cysts, fourteen (56%) underwent spontaneous regression by 6-8 months. Eight were operated in newborn period while 3 were operated in early infancy. Seven had ovarian cyst on right side, 4 had on left side. Eight babies underwent laparoscopy while 3 underwent laparotomy. Histopathology showed varied features of hemorrhagic cyst with necrosis and calcification, serous cystadenoma with hemorrhage, benign serous cyst with hemorrhage and simple serous cyst. Post-operative recovery was uneventful in all.

Conclusion: All the ovarian cysts detected antenatally in female fetuses need close follow-up after birth. Since spontaneous regression is known, only complex or larger cysts need surgical intervention, preferably by laparoscopy. Majority of the complex cysts show atrophic ovarian tissue hence end up in oophorectomy but simple cysts can be removed preserving normal ovarian tissue whenever possible.

## INTRODUCTION

Excessive stimulation of the fetal ovary by both placental and maternal hormones may lead to cyst formation [1]. The incidence of antenatally diagnosed ovarian cysts is 1 in 2500 live births [2-4]. Occasionally ovarian cysts may lead to several complications such as hemorrhage, rupture, torsion, bowel obstruction, necrosis, compression of the urinary tract, compression of the vena cava, hydramnios, and even cyst incarceration in the canal of Nuck [5]. Torsion is the most common complication in larger cysts [5-8].

Large and complex cysts are managed by surgical intervention while asymptomatic, simple or uncomplicated cysts, less than 5cm in size, can be observed, with regular monitoring of the cyst size using ultrasound until it resolves completely which usually takes 6-10 months [1-18]. Depending upon clinical features or postnatal ultrasound, surgery can be done in the newborn period or later when cysts becomes symptomatic [10-13].

In the present study, we have collected data regarding the clinical features, radiological findings, pathology and mode of treatment of ovarian cysts in female neonates.

## MATERIALS AND METHODS

Over a period of 6 years between 2009 to 2015, we followed-up 25 female newborns with antenatally detected intra-abdominal cysts which were confirmed by post-natal ultrasound or computerized tomography (CT) scan when indicated, as ovarian cysts. Alpha-feto protein and Beta-human chorionic gonadotropin (HCG) were estimated when complex cysts were seen on imaging. Babies with simple cysts, less than 5cm, were observed while those with larger/complex cysts underwent surgery. Clinically stable patients were subjected to laparoscopic procedures while the rest underwent laparotomy. Attempts were made to preserve normal ovarian tissue on the ipsilateral side whenever possible. Laparoscopy was intiated by Hassans technique with pressure of of 5-6 mmHg, with flow rate of 1-1.5Lts/minute; working port size was of 3mm and camera port was 5 mm: 30-degree Telescope was used.

## RESULTS

Of the 25 cases, 11 (44%) babies underwent surgical treatment. Fourteen (56%) cases underwent spontaneous regression during follow-up, by 6-8 months of age. All the cysts which showed regression were simple cysts less than 5 cm in size on the initial postnatal ultrasound. Of the 11 newborns, 8 needed surgery soon after birth (Fig.1), 6 of them underwent laparoscopic procedure. Three babies who were observed initially in view of small sized cysts on the initial scan, were noticed to have developed complications on follow-up imaging and required surgery later (Fig.2); 2 of them underwent laparoscopy.

At surgery 7 had cysts in the right ovary, 3 had cysts in the left ovary and 1 baby had cyst in the left ovary with a right mesosalpingeal cyst. Per-operatively 4 had large hemorrhagic cysts and 4 had torsion of the ovary and all of them underwent oophorectomy. One baby had necrosed cyst wall adherent to adjacent small bowel but the bowel was salvaged during oophorectomy (Table 1). One other baby who had a large complex ovarian cyst also underwent oophorectomy. Four of these cases who had ipsilateral hypoplastic fallopian tube underwent salpingo-oophorectomy. Only one baby who had a simple serous cyst underwent cystectomy preserving normal ovarian tissue. Average hospital stay was 5-7 days. Post-operative period was uneventful.

Histopathology showed features of hemorrhagic cysts with necrosis and calcification, serous cyst-adenoma with hemorrhage, benign serous cyst with extensive necrosis, and simple serous cyst (Table 2). All these babies were doing fine on followed up for a mean period of 12 months.

**Figure:**
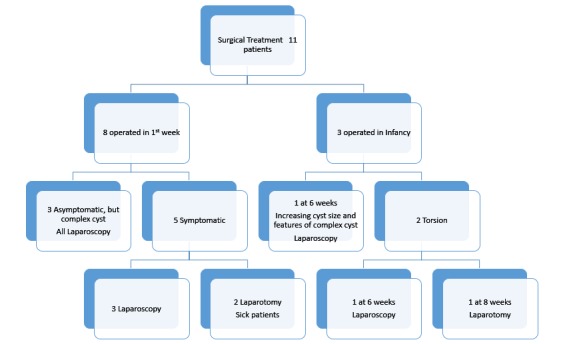
Figure 1: Treatment flow chart in our series

**Figure:**
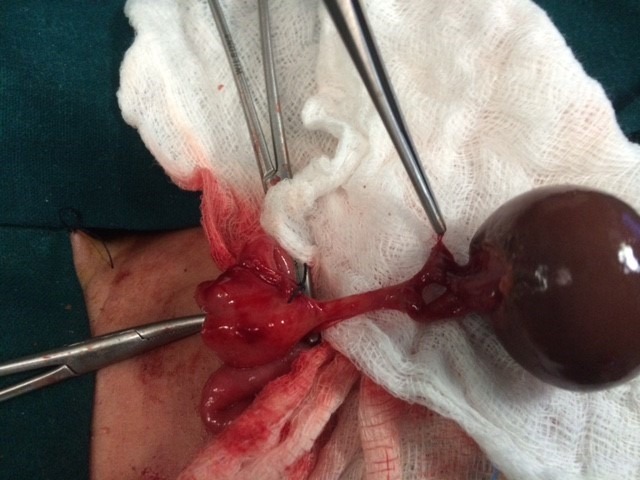
Figure 2: ovarian cyst torsion managed with open approach.

**Figure:**
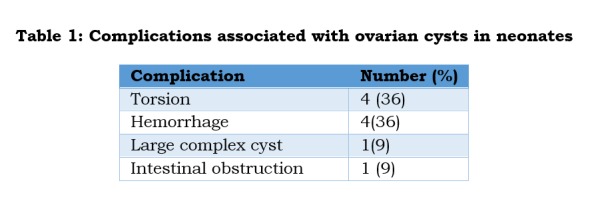
Table 1: Complications associated with ovarian cysts in neonates

**Figure:**
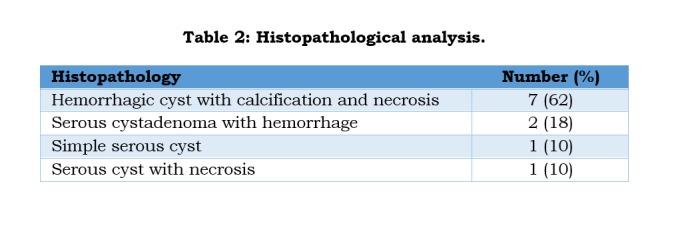
Table 2: Histopathological analysis.

## DISCUSSION

The most important aspect of laparoscopy management of ovarian lesions in neonates is proper instrumentations and proper case selection. We used 3mm working ports and 5mm camera port which were meant for neonatal laparoscopic surgery. Laparoscopy has the advantage of minimal invasive nature and prompt and good recovery in children and smooth postoperative and early smooth postoperative recovery. The treatment of ovarian cyst in the newborn is highly debated and there is no consensus on the management. The treatment of anechoic cysts depends on their size at diagnosis. Cysts that are less than 5 cm in diameter are unlikely to cause problems and can be observed for spontaneous resolution. Large cysts (>5 cm) have a high risk of complications (such as torsion, hemorrhage) and may present with pain, vomiting and abdominal distention. Hence, these cysts should be punctured or removed surgically [1,15]. At some centers, simple ovarian cysts are safely aspirated antenatally under ultrasound guidance [19]. 

In our series, fourteen neonates underwent spontaneous regression by 6-8 months; all 14 cases had cysts less than 5 cm in size. Eleven neonates underwent surgical treatment; 8 needed surgery soon after birth while three of our cases became symptomatic on follow-up and were operated at 1-2 months of age. Per-operatively, 4 cases had large hemorrhagic cysts, 4 had torsion of the ovary, one baby had necrosed cyst wall adherent to adjacent small bowel and one had a large complex cyst. All of them had ipsilateral oophorectomy and 4 of them had salpingo-oophorectomy (for hypoplastic fallopian tube). We could preserve the ovary in only one baby who had simple serous cyst and underwent cystectomy.

Most common complication of the ovarian cysts is torsion followed by hemorrhage which can be safely managed by laparoscopy in the present era of minimally invasive surgery, [2,4,5,9,10,12,13,17,19]. As the neonatal ovary has a long pedicle, the cyst after decompression can be easily delivered via umbilical port site and oophorectomy done. Cystectomy (ovary preserving) can be performed intra-corporally. In addition, the opposite ovary can be observed clearly [5,10,12]. Intestinal obstruction though uncommon is not a rare presentation of complex ovarian cysts and quiet often needs surgical intervention [6,8,14,16]. In our series, 8 cases underwent laparoscopic procedure of which 6 were newborns. Histopathological findings are simple serous cyst with hemorrhage, or necrotic tissue with no normal ovarian tissue in cases of torsion. Serous cystadenomas, teratoma and malignant ovarian tumors in newborns are rarely seen.

## CONCLUSION

Antenatally diagnosed intra-abdominal cysts in female fetuses should be followed-up immediately postnatally as there is great potential of resolution especially in simple cysts with diameter less than 5mm. Large Ovarian cysts or complex cysts should undergo surgery at the earliest. Neonatal laparoscopy is a safe option and should be recommended whenever possible. Preservation of normal ovarian tissue should be attempted whenever it is feasible.

## Footnotes

**Source of Support:** None

**Conflict of Interest:** None
